# Manufacturing Different Types of Solid Dispersions of BCS Class IV Polyphenol (Daidzein) by Spray Drying: Formulation and Bioavailability

**DOI:** 10.3390/pharmaceutics11100492

**Published:** 2019-09-25

**Authors:** Gean Pier Panizzon, Fernanda Giacomini Bueno, Tânia Ueda-Nakamura, Celso Vataru Nakamura, Benedito Prado Dias Filho

**Affiliations:** 1 Post-graduate Program in Pharmaceutical Sciences, Laboratory of Technological Innovation in the Development of Drugs and Cosmetics, State University of Maringá, Maringá 87020-900, Paraná, Brazil; gean_panizzon@yahoo.com.br (G.P.P.); tunakamura@uem.br (T.U.-N.); cvnakamura@uem.br (C.V.N.); 2 Medical and Pharmaceutical Sciences Center, Western Paraná State University, Cascavel 85819-110, Paraná, Brazil; buenofgb@gmail.com

**Keywords:** daidzein, solid dispersion, polyphenols, bioavailability enhancement, spray drying

## Abstract

Daidzein (DZ) is a polyphenolic compound belonging to Biopharmaceutical Classification System class IV, which shows that it may have limited therapeutic effects due to its low solubility and poor bioavailability. This study aimed to obtain high-purity DZ and prepare and characterize different types of solid dispersions (SDs) in order to enhance aqueous solubility and bioavailability. Excipients were investigated in order to manufacture different types of solid dispersions (SDs). Second-generation solid dispersions (SG), third-generation solid dispersions (TG), and second- and third-generation pH-modulated solid dispersions (SD and TG pHM-SD) were produced via spray drying. The SDs were characterized and tested for in vitro DZ release and oral bioavailability. SDs have shown increased aqueous solubility and in vitro release rate. Solid-state characterization showed that DZ was in an amorphous state in most of the formulations. The enhanced aqueous solubility of TG-pHM SD was reflected by an increase in oral bioavailability, which significantly increased the maximum plasma concentration approximately 20-fold and decreased the time to reach the maximum plasma concentration. The production of pHM SDs that contain DZ via spray drying is a simple and effective approach for oral drug delivery, which has the potential to greatly reduce the dose and enhance therapeutics effects.

## 1. Introduction

**Figure 1:**
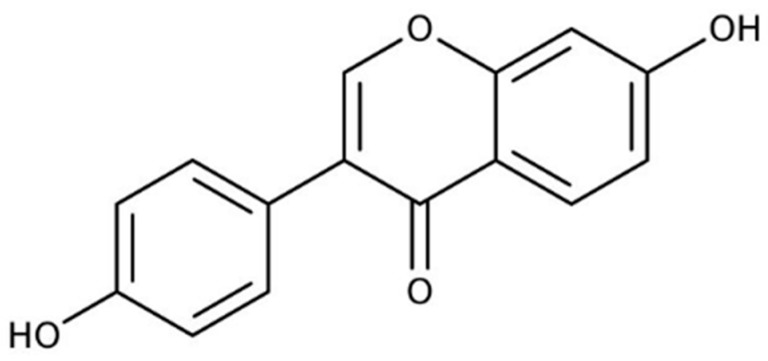
Chemical structure of Daidzein.

In recent years, there have been many studies on Daidzein (DZ), which is a soybean isoflavone aglycone. Biological effects of DZ include the prevention of osteoporosis [1] and breast cancer [2]. It also has neuroprotective [3] and cardioprotective effect [4], in addition to reducing hyperglycemia [5]. Thus, numerous DZ-based dosage-form formulations and dietary supplements have been produced worldwide, considering that most of them are polyphenols belonging to Biopharmaceutical Classification System (BCS) class II or IV. DZ is a weak acid (pKa = 9.6 for the hydroxyl group at position 4′ and pKa = 7.50 at position 7) with log*P* 2.51 [6] and high crystallinity [7] (Figure 1).

Daidzein’s low aqueous solubility and permeability make it a member of BCS class IV [8] and limit its bioavailability and biological effects [9]. Drugs with these physicochemical characteristics have limited applications in nutraceutical and dosage-form formulations, and technological strategies are required to overcome oral delivery limitations. Solid dispersions (SDs) are defined as dispersions of a drug into a solid-state inert matrix. They have been considered the most successful strategy for enabling drug solubility and bioavailability [10]. According to their composition, SDs can be classified as first generation, in which carriers and drugs are in a crystalline state [11]. This state is more thermodynamically stable than amorphous SDs. Second-generation SDs (SG) employ amorphous polymers and the drug may be molecularly dissolved or dispersed. Third-generation SDs (TG) have similar characteristics to SG, but a surfactant is added to the system to increase the drug dissolution and reduce problems of precipitation and recrystallization [11].

Recently, agents that are able to generate a modulated pH in the microenvironment and change drug release from solid oral dosage forms have been added to SDs [12]. Since then, pH-modulated SDs (pHM-SD) have been explored as an effective alternative to overcome the solubility and bioavailability issues of weak-acid and weak-base drugs [13]. Such pHM-SD can be broadly employed because almost two-thirds of substances with low solubility are weak acids or bases with pH-dependent solubility [12]. The most suitable excipients for producing SDs are the ones that have better miscibility and drug affinity. Consequently, such selection needs to be optimized by screening procedures [13]. Food and pharmaceutical industries commonly use spray drying in various applications. Spray drying of poorly water-soluble drugs is mainly aimed at generating amorphous materials and is the technique of choice for particle size reduction [10], which is suitable for the small-scale manufacturing methods or early development and also for the large-scale production of formulations of SD dosage forms [14]. Some studies have assessed the enhancement of DZ solubility by SDs [15]. Later, Feng et.al [16] reported the enhancement of DZ bioavailability by the SDs obtained with polyvinylpyrrolidone.

Although these studies have shown the feasibility of obtaining only SG with daidzein, none of them evaluated different types of SDs. Therefore, in this study, we purified, produced, and assessed four types of DZ SDs, including SG, TG, SG-pHM, and TG-pHM SDs using the spray-drying technique and optimized excipients. We also assessed their physicochemical characteristics through a scanning electron microscopy (SEM), differential scanning calorimetry (DSC), X-ray powder diffraction (XRPD), and gel permeation chromatography (GPC). We have also determined an in vitro release profile using a flow-through cell apparatus (USP apparatus IV). Finally, the pharmacokinetic profile of purified DZ and optimized SDs was compared in rats using a validated high-performance liquid chromatography–tandem mass spectrometry (HPLC-MS/MS) method.

## 2. Materials and Methods

Analytical standard of DZ, genistein and glycitein (IS; internal standard) were purchased from Tecpar (Curitiba, Brazil; purity ≥98%). Soybean extract (~20% of DZ) was purchased from Galena^®^ (Campinas, Brazil). Polyvinylpyrrolidone K90 (PVP), benzalkonium chloride (BAK; Fluka^®^, ≥95%, Milwaukee, WI, USA), sodium dodecyl sulfate (SDS), and Type HP-2 β-glucuronidase/sulfatase from Helix pomatia (197114 units/mL β-glucuronidase; 876 units/mL sulfatase) were purchased from Sigma-Aldrich^®^ (St Louis, MO, USA). Purified water was supplied by a Milli-Q system (Millipore Co., Bedford, MA, USA). We have also used acetonitrile, acetic acid (HPLC grade; J.T. Baker Chemical Co., Phillipsburg, NJ, USA), dimethyl sulfoxide (Sigma-Aldrich^®^), Rompun^®^ (2%, Bayer), Ketamine Agener (10%, Agener União, São Paulo, Brazil) and Na_2_CO_3_ (Merck, Darmstadt, Germany). All other used reagents and solvents were of analytical grade and were used as received.

### 2.1. Extraction and Purification of DZ

The purification of DZ from soybean extract was carried out in accordance with Zhang et al. [17] with modifications. Soybean extract (50 g) was dispersed in 0.1% (*v*/*v*) acetic acid (1 L) and kept under magnetic stirring (1 h, 1000 rotations per minute [rpm], C-Mag HS, Ika, Germany). The dispersion was then centrifuged (4 × 30 min, 15,000 g, 4 °C, Optima LK-90, Beckman Coulter, Krefeld, Germany), the supernatant was discharged and the slurry was freeze-dried (Alpha-2, Martin Christ, Osterode am Harz, Germany). Then, freeze-dried slurry was dispersed in 20% EtOH (*v*/*v*) and kept under magnetic stirring (1 h, 1000 rpm). The supernatant was discharged, the EtOH excess was removed by rotavapor (r-200, Buchi, Flawil, Switzerland), and the slurry was freeze-dried. We have carried out twice the dispersion, rotavaporing and freeze-drying with EtOH. The freeze-dried material was added to 2 L of anhydrous EtOH, kept under magnetic stirring for 4 h (1000 rpm, 80 °C), and vacuum-filtered (0.45 µm). In the end, anhydrous EtOH was removed under reduced pressure (MiVac Quattro, Genevac, Ipswich, UK).

### 2.2. Solubility of DZ and Screening of Excipients for SD Manufacturing

The drug solubility was evaluated in water, buffered solutions (pH 1.2–10; USP, 35), or a binary system of EtOH and water (20–80% EtOH, *v*/*v*). The screening of excipients was evaluated with 10 hydrophilic polymers, six surfactants, and four alkalizers at three different concentrations (0.5%, 1%, and 2%, *w*/*v*). Excess DZ (20 mg) was added to 1.5 mL of each solution. The samples were subjected to constant shaking in a metabolic bath (48 h, 50 rpm, 37 ± 0.5 °C, MA093, Marconi), centrifuged (5000× g, 15 min, Hettich Mikro 220, Tuttlingen, Germany), filtered (0.22 µm), diluted, and analyzed by HPLC (Waters, Alliance 2695 equipped with photodiode array detector Waters 2998, Milford, MA, USA). Empower software (version 3) (Milford, MA, USA) was used for instrument control and data acquisition.

### 2.3. Manufacturing of SDs by Spray Drying Technique

Table 1 shows the experimental design for manufacturing SG, TG, SG-pHM, and TG-pHM SDs.

In order to manufacture SG, we have dissolved PVP and DZ in 80% EtOH (*v*/*v*) using an ultrasonic bath (40 °C, 1 h, Unique, Brazil). To manufacture TG, SDS was also added. To manufacture SG-pHM and TG-pHM SDs, we have initially dissolved Na_2_CO_3_ and DZ in purified water so that PVP and (in the case of TG-pHM) SDs were dissolved under moderate magnetic stirring (30 min, 1500 rpm). A pilot spray dryer (model LM MSD 1.0, Labmaq, Brazil) was used. The following conditions were used: 0.7 mm pressure atomizer nozzle, feed solution flow rate of 6 mL/min, 40 L/h atomizing air flow, three-bar air pressure, 110 ± 2 °C inlet temperature, and 80 ± 2 °C outlet temperature. The spray dryer process parameters (i.e., inlet temperature, airflow rate, feed solution flow rate, and feed concentration) were selected by assessing the yield, morphology, and glass transition temperature (Tg) of the polymer.

### 2.4. Saturation Solubility

Saturation solubility of purified DZ, physical mixtures (PMs), and spray-dried formulations were determined by the shake flask method. Excess DZ (25 mg), corresponding to the weight of the SDs and PMs containing the same proportions of each component, were added to 1.5 mL of purified water. The samples were subjected to the same procedure as described in solubility assay and then diluted and analyzed by HPLC (Waters, Alliance 2695) equipped with photodiode array detector Waters 2998. Empower software (version 3) was used for instrument control and data acquisition

### 2.5. Characterization of Solid Dispersion

Loading efficiency (LE), SEM, and particle size measurement have been carried out in accordance with Panizzon et al. [18] with modifications. The amount of DZ was determined by using HPLC. Analyses were carried out on a Waters 2695 Alliance (Milford, MA, USA) equipped with PDA detector (Waters 2998), and software Empower 3 (Milford, MA, USA). The used mobile phase was: Channel A: 0.1% acetic acid in 18.2 MΩ/cm water (solution A) and Channel B: 0.1% acetic acid in acetonitrile (solution B). The used gradient in Channel B was: 14% (8 min), 14% to 21% (4 min), 21% (3 min), 21% to 29% (5 min), 29% to 40% (3 min), 40% to 50% (2 min), 50% (5 min), 50% to 14% (5 min), 14% (5 min). The injection volume was 20 µL and the UV–Vis detection system was monitored at 254 nm. The flow rate was 1.0 mL/min and the temperature was 30 °C. The separation was carried out on a Gemini C18, 250 × 4.6 mm, 5-µm column (Phenomenex Inc., Torrance, CA, USA). We have placed the samples in tubes containing acetonitrile (10 mL) and shaken them (500 rpm × 5 min). Distilled water (10 mL) was added and shaken again for 60 min. The mixture was filtered through a 0.22 µm membrane and injected.

Morphology and mean particle size were examined by SEM (Shimadzu SS-550, Kyoto, Japan). The material was fixed on double-sided tape attached to an aluminum support, then coated with gold/palladium under argon atmosphere, and examined under a scanning electron microscope. The mean particle size was determined with the aid of an Image-Pro^®^ Plus image analyzer (Silver Spring, MD, USA), by measuring the Ferret’s diameter of at least one thousand particles present in the photomicrographs. DZ content (DZC), loading efficiency and span were calculated by using the following equations (1)–(3):

DZC (mg/g) = weight of DZ in formulation/weight of formulation(1)

Loading efficiency (%) = (weight of DZ/theoretical weight of DZ) × 100(2)

Span = (*d*(0.9) − *d*(0.1))/*d*(0.5) (3)

### 2.6. X-ray Diffraction (XRPD)

X-ray Diffraction patterns have been obtained by using an X-ray diffractometer (Bruker-AXS, Karlsruhe, Germany). The specifications were 2°/min, 2*θ*, scanning from 10° to 70°, Cu-Kα X-radiation (*λ* = 1.5418 Å), 40 mA current, and 40 kV voltage.

### 2.7. Differential Scanning Calorimetry (DSC)

DSC analyses have been carried out by using a calorimeter (Q20, TA instruments, New Castle, DE, USA) operating at the following conditions: heating rate of 10 °C/min, nitrogen flow rate of 50 mL/min, and temperature range from 25 to 350 °C. Samples (6–9 mg) were placed on an aluminum DSC pan and hermetically sealed with a lid (Tzero^®^, TA instruments, New Castle, DE, USA). An empty pan was used as a reference. Indium standard was used to calibrate the DSC temperature and enthalpy scale. Data acquisition and analysis were carried out by using Advantage software v.5.22 (TA Instruments, New Castle, DE, USA).

### 2.8. Gel Permeation Chromatography (GPC)

The molecular weight profile changes of PVP were determined by GPC on a Waters Alliance 2695 (Waters) equipped with an evaporative light scattering detector Waters 2424. Data analyses were carried out in Empower version 3 (Waters). Gel permeation chromatography columns: Ultrahydrogel-500, Ultrahydrogel-250, and Ultrahydrogel-120 (Waters) were connected in order to decrease pore size and maintained at 45 °C. The detector nebulizer was tuned in cooling mode, the drift tube temperature was kept at 80 °C, and the nitrogen pressure was kept at 50 psi. The used mobile phase was acetonitrile:water (20:80, *v*/*v*), with a flow rate of 0.8 mL/min and an injection volume of 10 µL. Molecular weight calibration was carried out by using Pullulan Calibration Kit (Shodex standard, Showa Denko, Knagawa, Japan) standards with a molecular weight range of 21.7–3050 kDa.

### 2.9. Dissolution Profile, Daidzein-Release Mechanism, and Kinetics

The in vitro dissolution profile of DZ and SDs was determined by using flow-through cell (USP Apparatus 4) (EC 7smart, Sotax Co., Aesch, Switzerland) on open mode. Twenty milligrams of DZ, calculated from the LE results, were added between the layers of glass beads (2 × 1 g; 1 mm) in the equipment cells. A glass microfiber filter (GF/B, Whatman, Maidstone, England, 1 µm pores) was coupled to the cells in order to prevent the passage of undissolved particles. Phosphate buffer (75 mM, pH 6.8) (USP 35) was used as a dissolution medium (4 mL/min, 37 ± 0.5 °C) and after 5, 15, 30, 60, 90, and 150 min, and aliquots of each sample were collected and analyzed by HPLC. The pH of the dissolution medium was monitored immediately after every sampling time using the pH meter (DM-22, Digimed, São Paulo, Brazil). The samples were filtered (0.22 µm), diluted, and analyzed by HPLC. Results have been reported as cumulative percentage of DZ dissolved in the medium over time. The DZ-release mechanism and kinetics SDs have been carried out in accordance with the Panizzon et al. method [18].

### 2.10. Pharmacokinetic Studies

The State University of Maringá Animal Ethics Committee approved the following procedures and protocols on 7 June 2011 under 043/2011 project identification code. Twenty-four male Wistar rats (*Rattus norvegicus*) at the age of 90 days were divided into two groups and kept under controlled conditions in a 12 h light/dark cycle at 22 ± 2 °C. We have provided them with water and a standard rodent chow (Nuvital^®^) diet ad libitum. Seventy-two hours before the treatment, soy-free chow was supplied and maintained ad libitum until 14 h before the treatment. Twenty-four hours before the treatment, rats were anesthetized (ketamine:Rompun^®^ 1:1, *v*/*v*, 1 mL/kg) and underwent surgery. A silicone cannula was implanted into the left jugular vein and stabilized on the dorsal region of the neck. Then, 10 mg of DZ and 0.73 g of F10 was suspended in 0.98 mL and 0.6 mL of olive oil, respectively. The final concentration of drug was 10 mg/mL. A dose of 10 mg/kg was administrated by gavage. Blood samples (300 µL each sample, total blood volume collection of 1.8 mL per animal) were collected at 0.083, 0.17, 0.25, 0.5, 1, 1.5, 2, 4, 6, 8, 12, and 24 h (*n* = 6) through the cannula and mixed with heparinized saline (50 µL). The volume of collected blood was replaced by sterile saline. Plasma was obtained by centrifugation (2500× g, 7 min) and stored frozen at −80 °C until analysis.

HPLC-MS/MS analyses were carried out with a Waters 1525µ coupled to a Micromass Quattro micro™ API triple-quadrupole mass spectrometer (Waters) with an electrospray ionization interface under the following conditions: capillary voltage of 2.5 kV (positive mode), cone voltage of 30 V, extraction cone voltage of 2 eV, source temperature of 130 °C, desolvation temperature of 450 °C, cone gas flow of 50 L/h, and desolvation gas flow of 750 L/h. The spectrometer was tuned in the multiple reaction-monitoring mode to monitor mass transitions m/z 254.94→90.83 (DZ) and m/z 285.91→270.87 (IS) with collision energies of 35 eV and 25 eV, respectively. MassLynx™ software (version 4.0, Waters) was used for data processing. The used column was Luna C18(2)-HST (2.5 µm; 50 × 2 mm, Phenomenex, Torrance, CA, USA), and the mobile phase consisted of water with 0.1% (*v*/*v*) formic acid (solvent A) and acetonitrile with 0.1% (*v*/*v*) of formic acid (solvent B) delivered at a rate of 0.3 mL/min, as follows: 5% solvent B at 0–0.5 min, 75% solvent B at 4 min, and 5% solvent B at 5 min. In order to determine total DZ (free and conjugated DZ), samples were enzymatically hydrolyzed as Qiu et al. method [9] with modifications. The enzyme β-glucuronidase (100 µL, 9000 U/mL in 0.05 mol/L acetate buffer, pH 5.0) was added to plasma (100 µL) and incubated at 37 °C for 14 h. An internal standard (glycitein, 35 µL; 1000 ng/mL) and purified water (2 mL) were added and samples were loaded onto a solid phase extraction cartridge C18 Amprep (500 mg, Amersham Pharmacia Biotech). The cartridge was flushed with acetic acid 0.1% (3 mL, *v*/*v*) followed by 25% methanol (3 mL) containing 0.1% (*v*/*v*) acetic acid. The analytes were eluted with methanol (2 × 3 mL) and evaporated to dryness in a MiVac Quattro Concentrator (Genevac, Ipswich, UK). The residue was reconstituted with 80% methanol (100 µL, *v*/*v*), and 10 µL was injected. The method was validated using bio-analytical method validation guidance (FDA, 2001) (Supplementary Materials). Pharmacokinetic analyses have been carried out by using a non-compartmental model. The maximum plasma concentration (Cmax) and time to reach the maximum plasma concentration (Tmax) were directly obtained from the mean blood concentration-time curve. The pharmacokinetic parameters were calculated by using Excel 2013 software. The areas under the concentration vs. time curves (0–24 h [AUC0–24 h] and 0 to infinity [AUC0→∞]) were calculated by using the trapezoidal method.

### 2.11. Statistical Analysis

The software Statistica^®^ 8.0 (StatSoft, Inc. 1984–2007, Tulsa, OK, USA) was used for statistical analysis. Data are presented as mean ± standard deviation (S.D.) using a unilateral analysis of variance (one-way ANOVA). Significant differences were determined with the Tukey test, with *p* < 0.05 considered statistically significant.

## 3. Results

### 3.1. Extraction and Purification of DZ

The overall recovery of DZ throughout the entire purification process was 63–69%. The final content, determined by HPLC, was 95.6% ± 1.4% DZ and 1.2% ± 4.8% genistein. Figure S1 shows the characterization of the material that was performed by using HPLC-MS/MS operating in full scan mode. The identity was confirmed in daughter scan mode by comparing the ion products (MS/MS) with published data [19]. The SEM and XRPD results showed that purified DZ was in a crystalline form, which was then used as a polyphenolic BCS Class IV drug model for DZ SD manufacturing.

### 3.2. Effect of Excipients and Solvents on DZ Solubility

**Figure 2:**
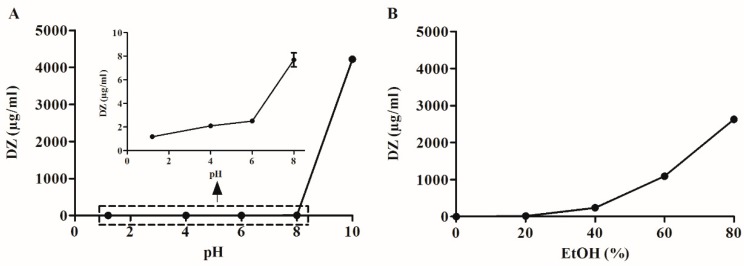
Solubility of Daidzein (DZ) in different solvents: (**A**) pH 1.2–10 buffered solutions, (**B**) water and hydroethanolic solvents (EtOH 20–80%, *v*/*v*).

Daidzein can be classified as practically insoluble in water (1.50 µg/mL). As expected for a weak acid, DZ has high solubility in basic pH in contrast to neutral and acidic pH (Figure 2).

This shows that DZ solubility is pH-dependent, and alkalinizers would be the best choice to prepare pHM SDs. Table SI shows the DZ-solubilizing capacity of 10 polymers, six surfactants, and four alkalinizers at different concentrations (0.5, 1, and 2%, *w*/*v*). Among the investigated polymers, PVP K90 was the most effective. BAK is a surfactant with a higher drug-solubilizing capacity, but SDS was chosen due to its lower toxicity [20]. Na_2_CO_3_ provided the highest amount of dissolved DZ and was used as an alkalinizer to prepare SG-pHM and TG-pHM SDs. Furthermore, water that contained 0.5% (*w*/*v*) Na_2_CO_3_ could replace 80% EtOH (*v*/*v*) as the solvent.

### 3.3. Manufacturing and Characterization of SDs

Daidzein (2.5 g/L) was dissolved in 80% EtOH (*v*/*v*) for the preparation of SG (F1 and F2 formulations) and TG (F3 and F4 formulations). DZ was dissolved in water containing 0.5% Na_2_CO_3_ (*w*/*v*) for the preparation of SG-pHM SDs (F5 and F6 formulations) and TG-pHM SDs (F7, F8, F9, and F10 formulations). Table 2 shows the DZ content, LE, mean particle size, and span. The LE was ≥ 95.6%. The mean particle size and span ranged from 2.48 to 7.09 µm and from 1.13 to 3.88, respectively.

**Figure 3:**
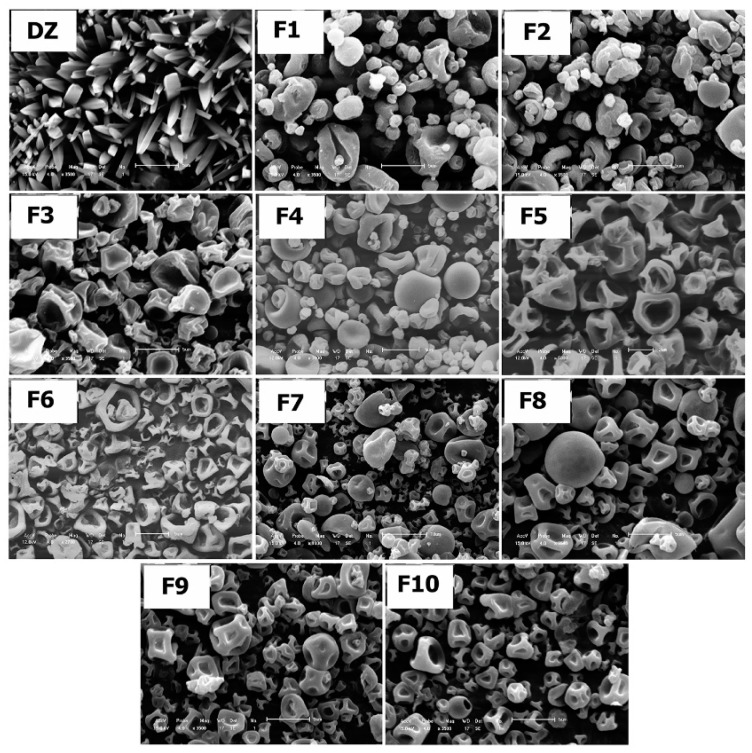
Scanning electron microscopy micrographs of Daidzein (DZ) and SDs (F1–F10). Scale bar in DZ, F1–F4, F6, F8–F10 is 5 µm; scale bar in F5 is 2 µm; scale bar in F7 is 10 µm.

Micrographs of purified DZ and SDs are shown in Figure 3. Drug crystals showed a prismatic shape with similar sizes. However, we could not note drug crystals in the SDs. The morphological results of all of the SDs were similar in terms of shape and matrix distribution. SDs had a smooth surface and concave depressions (collapsed walls), with no agglomerates or pinholes.

### 3.4. Saturation Solubility

**Figure 4:**
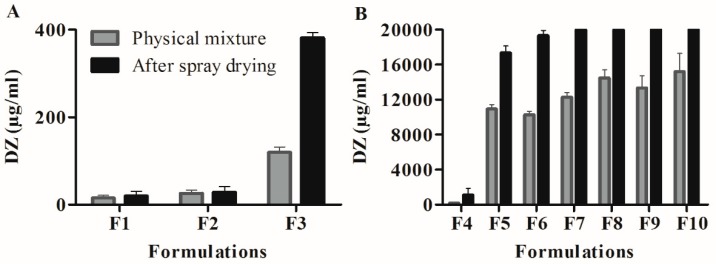
Saturation solubility of Daidzein (DZ) in physical mixtures (PM) and solid dispersion (SD): (**A**) F1–F3 and (**B**) F4–F10 (mean ± S.D, *n* = 3).

The results of the saturation solubility assay are shown in Figure 4. When the results were compared with the PMs, only the SG was unable to significantly increase the water solubility of DZ (*p* > 0.05). The TG, SG-pHM, and TG-pHM SDs resulted in significant increases in the solubility of DZ (*p* < 0.05). The pHM SDs showed the highest capability of solubilizing high doses of DZ (>20 mg/mL).

### 3.5. Differential Scanning Calorimetry Analyses

**Figure 5:**
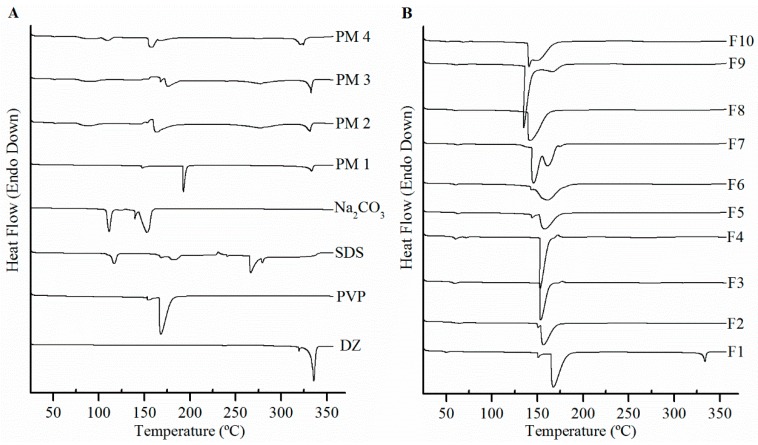
Differential scanning calorimetry (DSC) thermograms of (**A**) daidzein (DZ), polyvinylpyrrolidone K90 (PVP), sodium dodecyl sulfate (SDS), sodium carbonate (Na_2_CO_3_), physical mixtures of DZ:PVP 1:1 (PM 1), DZ:PVP:SDS 1:1:1 (PM 2), DZ:PVP: Na_2_CO_3_ 1:1:1 (PM 3) DZ:PVP:SDS: Na_2_CO_3_ 1:1:1:1 (PM 4), and (**B**) DSC solid dispersions (F1–F10).

The DSC thermograms of DZ, excipients, PMs, and spray-dried formulations (F1–F10) are shown in Figure 5A,B. The DSC results showed no phase transition of the drug until the melting point of genistein (319.3 °C) and DZ (336 °C), where two sharp endothermic peaks that are characteristic of the crystalline solid state were found. The DSC trace of PVP K90 showed endothermic peaks at 153 and 168 °C. Conversely, there was no noticeable peak at melting point of DZ in any of the excipients and PM. The melting peak of crystalline DZ were observed in all PM. Thus, DSC results could be used to evaluate solid state of DZ. With the exception of F1, the DZ melting peak disappeared completely in all other formulations.

### 3.6. X-Ray Powder Diffraction Analyses

**Figure 6:**
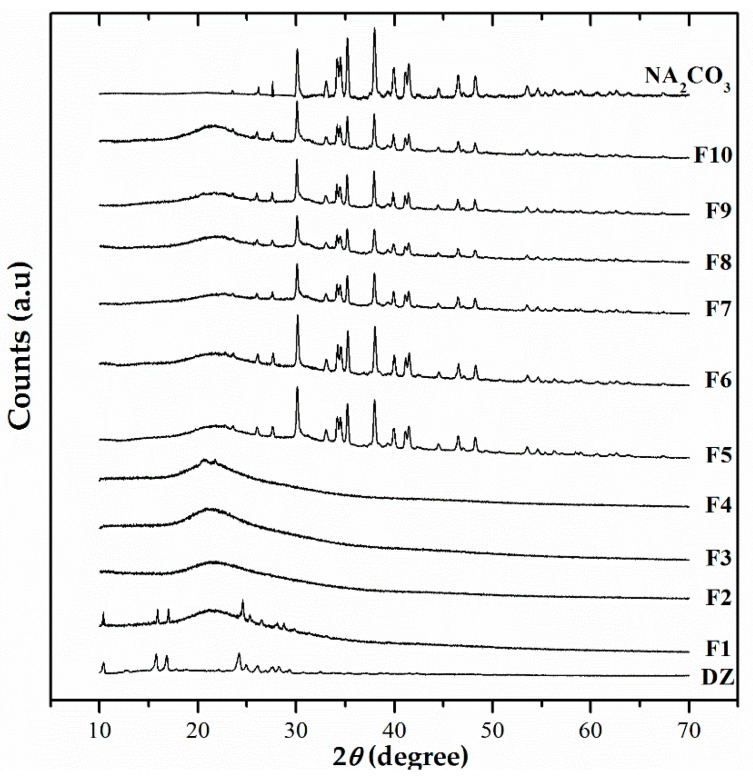
X-ray powder diffraction patterns of DZ and spray-dried formulations (F1–F10).

Figure 6 shows the XRPD patterns of purified DZ, formulations, and Na_2_CO_3_. The XRPD analysis showed that DZ was a crystalline powder with sharp peaks at 2*Ɵ* equal to 10.4°, 15.8°, 16.8°, 24.2°, 24.9°, 26.1°, and 28.2°. Among the excipients, only Na_2_CO_3_ was crystalline and responsible for the diffraction peaks shown in F5 to F10. The F1 diffraction pattern showed that DZ was still present in its crystalline state, probably due to the low concentration of the polymer. F2, TG, SG-pHM, and TG-pHM had patterns that were analogous to the carriers.

### 3.7. Evaluation of PVP Structural Modification by Gel Permeation Chromatography (GPC)

The results showed that polymer Mw had no changes in SG or TG (Mw = 1200 kDa). However, the size changing for SG-pHM and TG-pHM (Mw = 2920 kDa) was shown by GPC.

### 3.8. Dissolution Profile

**Figure 7:**
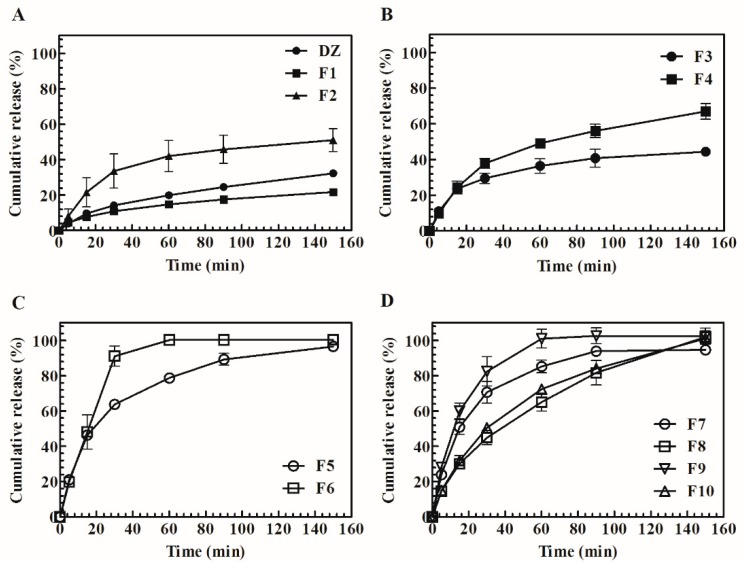
Cumulative release (%) of (**A**) Daidzein (DZ), SG (F1 and F2), (**B**) TG (F3 and F4), (**C**) SG-pHM (F5 and F6) and (**D**) TG-pHM (F7, F8, F9 and F10) (mean ± S.D, *n* = 3).

Daidzein precipitation and significant variations in pH (>0.1) were not noted in the collected samples. The dissolution profile of DZ showed a slow release rate, and F1 was unable to improve solubility (Figure 7A). Some enhancement in in vitro release was reached with a higher amount of the polymer (F2). A higher release rate was observed only when 10% SDS (*w*/*w*; F4) was added (Figure 7B), but it was still incomplete. The SG-pHM SD (Figure 7C) had faster and higher release rates compared with the previous SDs. TG-pHM SDs, with 1:2 and 1:4 DZ:polymer ratios, were also prepared (Figure 7D). The TG-pHM SDs effectively increased the release rate, even with lower PVP content (1:2 DZ:polymer ratio).

### 3.9. Daidzein-Release Mechanism and Kinetics

The coefficients of determination (R2) of power law best fit the in vitro release data, showing that DZ release was run by anomalous transport (0.43 < *n* < 0.85; Table SII). However, the diffusion process was predominant for F3 (*n* = 0.40) and super case II was predominant for F6 (*n* = 0.85).

### 3.10. Pharmacokinetic Studies

**Figure 8:**
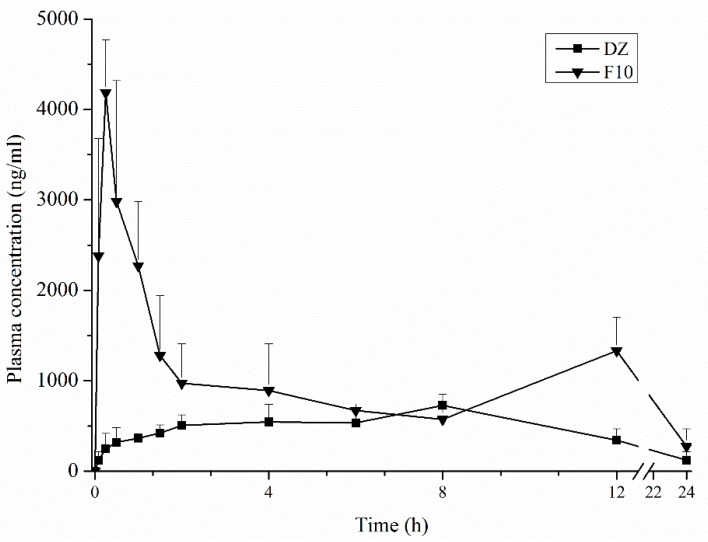
Mean plasma concentration–time curves of Daidzein (DZ) after oral administration of pure DZ (▬▄▬) and F10 (▬_▼_▬) (mean ± S.D, *n* = 6).

HPLC-MS/MS results of specificity (Figure S2), linearity, precision, accuracy, extraction recovery, and matrix effect (Table SIII) are shown in the Supplementary Information. Figure 8 shows the mean plasma concentration–time profiles of DZ following administration of a 10 mg/kg dose of optimized SD and DZ in rats. Two peaks of DZ occurred in plasma. The first peak of purified DZ occurred at 4 h, and the second peak occurred at 8 h, with low concentrations as reported in other studies [9]. For F10, the first plasma peak occurred at 15 min and the second peak at 12 h.

The Tmax of purified DZ and F10 was 6.5 ± 1.9 and 0.26 ± 0.1 h, respectively. Cmax of purified DZ and F10 was 707.2 ± 91.6 and 4050.50 ± 506.7 ng/mL, respectively. The AUC0–24 h and AUC0→∞ for purified DZ and F10 were 9143.3 ± 1577.8 and 21995.3 ± 3025.5 ngh/mL and 11442.2 ± 2201 and 28305.4 ± 4691.9 ngh/mL, respectively.

## 4. Discussion

Several studies have shown the biological effects of DZ, making it a promising compound for the treatment of several diseases [21]. Nevertheless, DZ is a BCS class IV polyphenol, with low aqueous solubility and low permeability, thus limiting its oral bioavailability. Solid dispersions are useful for increasing the aqueous solubility and bioavailability of drugs under these characteristics.

High-purity DZ was isolated from soybean extract using a modified method described by Zhang et al. [17]. In order to manufacture SDs by spray drying, it was necessary to test drug solubility in solvents that are able to dissolve both excipients and DZ. In order to manufacture SG and TG, 80% EtOH (*v*/*v*) was used due to its volatility, low toxicity [12,22] and ability to dissolve high amounts of DZ (2629 µg/mL). However, Na_2_CO_3_ had limited solubility in 80% EtOH (*v*/*v*). Consequently, Na_2_CO_3_ was dissolved in water (0.5% *w*/*v*) and then used to dissolve DZ. This solution was spray-dried to manufacture the pHM SDs.

In the development stage, the selection of excipients has a significant effect on the solid state and stability of the amorphous state under in vitro and in vivo conditions. Considering this, solubility tests were carried out with several polymers, surfactants, and alkalizers. The aim was to study and select the excipients by using a simple and effective approach, which is based on individual capability of dissolving the drug to obtain different generations of SDs [13,21,23].

The characterization of SDs showed that spray drying provided high drug LE. This may occur due to rapid solvent evaporation that led to a rapid increase in viscosity, thus permitting kinetic trapping of the drug in the matrix [10]. SEM analysis revealed needle-shaped DZ crystals. Crystal habit of drug was not found on SEM micrographs after spray drying. The SDs had similar morphology to PVP-containing microparticles that have been obtained by spray drying in a previous study [24]. The low viscosity of the solution was insufficient to maintain the physical stability of the droplets during solvent evaporation, which may have led to this characteristic shape [25]. After manufacturing the formulations, the influence of the spray-drying technique on the DZ aqueous solubility was studied by comparing the amount of DZ that was dissolved by formulations with the amount that was dissolved by the PMs in the solubility saturation tests. The results have shown that spray drying effectively increased DZ solubility compared with the PMs.

DSC and XRPD assessed DZ solid state. The DSC analyses revealed the absence of endothermic or exothermic peaks at DZ melting point of (336 °C), showing that there was no interference caused by the excipients or their mixture on DZ solid-state characterization in the formulations. DZ melting peak in PM showed that the solid state of drug had no alterations. The absence of a DZ melting peak may indicate the transformation of crystalline DZ into an amorphous form during the spray drying process, which would partially explain the enhancement in solubility [26]. The PVP used in this study presented two endothermic peaks at 154 and 168 °C. Although they are normal temperatures for Tg of PVP K90, this unusual DSC trace may be associated to changes in molecular weight, crystallinity degree, and more notably in purity of PVP obtained from different origins [27,28].

The XRPD analyses of the formulations are consistent with previous DSC data, showing that DZ has an amorphous solid state. The amorphous state consists of molecules that are randomly distributed with high free energy. Little or no energy is required to separate them, thus facilitating their dissolution [29] and improving their bioavailability [30].

Various excipients such as alkalinizing and acidifying agents and processes have been used to obtain amorphous SDs by the spray-drying technique [12,13,31]. However, little information about their influence on the polymeric carrier is available. The GPC technique was used to study the influence of process conditions and excipients on PVP Mw characteristics. PVP is a water-soluble polymer in a wide range of pH (1–10). However, under conditions of alkalinity and high temperatures, cross-linking may occur. Thus, PVP may become insoluble, depending on the extent of cross-linking, but it remains swellable [32]. PVP may be used for sustained or controlled release. When there is an increase in Mw, there is also a modification of the drug release rate [33]. This may be related to the slowest release rate of the TG-pHM SD with a higher polymer ratio (F8 and F10). Thus, during the development and manufacturing of pHM SDs, molecular changes in PVP should be considered to ensure that the drug release kinetics are not impaired.

The dissolution phase was performed under sink conditions by using a USP apparatus IV (flow-through cell). This apparatus is the most suitable pharmacopeical method for testing poorly water-soluble drugs and distinguishing between different formulations, thus decreasing the number of tests and animals in pharmacokinetic studies [34]. The results showed that the F1 release profile may be correlated with the crystalline state that was observed in the XRPD and DSC analyses. The highest polymer:drug ratio is known to have a beneficial influence on solubility and consequently drug release [35]. For this reason, for all of TG systems, a 1:4 DZ:polymer ratio was maintained, and the influence of SDS (5% and 10% *w*/*w*) was evaluated. Notably, surfactants might enhance drug wettability, decrease surface tension, trigger drug release from SDs, and prevent nucleation and crystal growth [36]. Moreover, SDS will be above the critical micelle concentration (CMC) during the drying stage of the spray drying. Above CMC concentration, the drug can be entrapped in different regions of the micelle and generally result in enhanced solubility of poorly soluble drugs [37].

These effects could be the reason for the improved results when 10% SDS was used. In recent studies, pHM SDs were successfully used to improve the aqueous solubility of poorly soluble ionizable drugs [12,13]. The in vitro release profile showed that SG-pHM SD (Figure 7C) had faster and higher dissolution rates. TG-pHM SD were assessed by adding a surfactant and with high drug loading (F7 and F9). Even under this condition, the formulations reached fast release, comparable to previous SDs. F8 and F10 had the highest DZ:polymer ratio (1:4) and reached some sustained release, which can be an advantage compared with SG-pHM SD. The in vitro drug release was not changed by a higher amount of SDS. In addition to the aforementioned factors, the enhancement of in vitro release by SDs may also be due to a decrease in particle size at the molecular level and decrease in agglomeration, porosity, and amorphization of the crystalline state [11]. The most important factor that controls drug release from SDs obtained with hydrophilic polymers is the establishment of a gel layer around the system where water is adsorbed [12]. At this site, release is caused by water infiltration, the swelling of polymers, drug dissolution and diffusion, and matrix erosion [34]. The drug is soluble and released into the dissolution medium until a saturation solubility limit is reached. Henceforth, dissolution occurs under non-sink conditions, leading to nucleation and crystal growth in the gel layer, the extent of which depends on the carriers and drug concentration.

Therefore, despite the apparent advantage of SG and TG, the generation of a supersaturated gel layer with a low threshold of saturation solubility can lead to insufficient oral bioavailability of the drug, which is likely to occur due to precipitation during release into the gastrointestinal tract [38]. Daidzein has pH-dependent solubility, and Na_2_CO_3_ allows the formation of a pH microenvironment with high saturation solubility threshold of the drug in the gel layer of SG-pHM SD. Furthermore, pHM SDs can release the drug regardless the pH of the environment [12,39]. The advantage of TG-pHM SD is the possibility to further increase the saturation solubility threshold in the gel layer due to presence of surfactant. TG-pHM SD carriers also play a crucial role in maintaining supersaturation and precipitation inhibition in vivo, which is widely accepted as critical in enhancing solubility in the gastrointestinal tract [40]. The release profiles generated by the flow-through cell shows that both pHM-SD systems can enhance DZ bioavailability.

Further characterization of the release kinetic profile, which is based on the data from the in vitro release tests, was performed by fitting the data to mathematical models, including zero and first orders, Higuchi, Hixson-Crowell, and Korsmeyer-Peppas (power law). These mathematical models can provide insights into the physical mechanisms and kinetics of drug release from SDs. Anomalous transport is a combination of Fickian diffusion and matrix swelling. The diffusion process was predominant for F3, which is likely to occur due to rapid elimination of the polymer that may be caused by its low amount and higher dissolution aided by the surfactant and causes consequent precipitation of the drug in the flow-through cell. In the super case II, the mechanism involves the relaxation of polymer chains and corresponds to zero-order kinetics. The release kinetics of SDs are controlled by the gel layer and depend on composition, stability, concentration, viscosity, and the chemical structure of the polymer [41].

In order to improve the oral absorption of BCS class IV polyphenol, we manufactured different types of SD systems and assessed DZ release. The characterization, saturation solubility, and in vitro release data were considered when choosing an optimized SD for comparisons with the oral bioavailability of purified DZ, thus requiring fewer animals. Formulations with high amount of PVP could be advantageous due to its mucoadhesive properties that are likely to help to enhance drug delivery [42]. The possibility to further increase the saturation solubility threshold in the gel layer, the role of Na_2_CO_3_ and SDS in maintaining supersaturation and precipitation inhibition in vivo [40] and the lack of data available about TG-pHM, made this an advantageous and interesting formulation to be evaluated in vivo. Based on this, we decided to performed pharmacokinetic study on F10.

In order to ensure the reliability of the results of the pharmacokinetic study, a HPLC-MS/MS method was validated. The plasma concentration profiles of DZ showed two peaks. The second peak is described in the literature and corresponds to the enterohepatic recirculation of DZ that was excreted in the bile and reabsorbed [43,44]. The plasma concentration profiles of purified DZ and F10 had different pharmacokinetics behavior. After oral administration of purified DZ, the plasma concentration increased slowly, unlike formulation F10. For F10, the first drug peak occurred at 15 min, representing a 20-fold increase at this time. Pharmacokinetic parameters (Cmax, Tmax, AUC0–24 h, and AUC0→∞) were significantly different between purified DZ and F10 (*p* < 0.05). For example, the Cmax and AUC0–24 h of F10 increased 5.7-fold and 2.4-fold, respectively, compared with purified DZ. These results show that F10 can improve bioavailability of drug.

Many efforts have been made to manufacture solid dosage forms with high bioavailability by changing the solid state [31]. The obtained F10 formulations by spray-drying technique also provided a higher Cmax and lower Tmax compared with DZ-loaded phospholipid complex polymeric nanoparticles and DZ-loaded cyclodextrin inclusion complex polymeric nanoparticles at the same dose [21,22]. The in vivo pharmacokinetic data have shown that the accelerated initial DZ release that was achieved by the spray drying technique and incorporation of excipients into SDs dramatically improved the extent of oral DZ absorption.

## 5. Conclusions

In this study, SG, TG, SG-pHM, and TG-pHM SDs that contained DZ were successfully manufactured by the spray-drying technique using PVP (K90), SDS, and Na_2_CO_3_ as the most appropriate excipients. All of the formulations had high DZ content, no detectable crystallinity, enhanced relative solubility, and an enhanced dissolution rate, with the exception of F1 formulation. PVP Mw may change based on the specific conditions. The results showed that a major obstacle when using amorphous SG and TG is the limited DZ solubilizing capacity. Thus, the pH-dependent solubility of DZ was reflected by the dissolution rate, and only the pHM SDs reached complete dissolution even with high DZ loading. The potential of TG-pHM SDs to increase solubility in the gastrointestinal tract and enhance absorption was reflected by enhanced oral bioavailability, which was shown by the pharmacokinetic analyses (i.e., increases in AUC and Cmax and a decrease in Tmax). TG-pHM SD formulation has the potential to enhance the therapeutics effects of DZ by increasing its absorption, thus broadening the therapeutic applications of such drugs. These results also provide an experimental basis for using spray-dried pHM SDs that contain poorly water-soluble ionizable polyphenols and drugs as a feasible drug delivery system to provide immediate release. Overall, it is an attractive alternative to enhance the therapeutic effects of polyphenolic drugs belonging to BCS class IV.

## Figures and Tables

**Table 1:** Experimental design for manufacturing solid dispersions (SDs).

SDs	DZ (g)	PVP (g)	SDS (%)	Na_2_CO_3_ (g)	Solvent	Volume of Solvent (mL)
**2nd generation (SG)**						
**F1**	2.5	5	-	-	80% EtOH	1000
**F2**	2.5	10	-	-	80% EtOH	1000
**3rd generation (TG)**						
**F3**	2.5	10	5	-	80% EtOH	1000
**F4**	2.5	10	10	-	80% EtOH	1000
**2nd generation + pH-modulated (SG-pHM)**						
**F5**	2.5	5	-	5	Water	1000
**F6**	2.5	10	-	5	Water	1000
**3rd generation + pH modulated (TG-pHM)**						
**F7**	2.5	5	5	5	Water	1000
**F8**	2.5	10	5	5	Water	1000
**F9**	2.5	5	10	5	Water	1000
**F10**	2.5	10	10	5	Water	1000

**Table 2:** Daidzein content (DZC), loading efficiency (LE), mean particle size, and span of solid dispersions (SD) F1–F10. The results are shown as mean ± S.D.

Parameter	SG		TG		SG-pHM		TG-pHM			
SD	F1	F2	F3	F4	F5	F6	F7	F8	F9	F10
**DZC (mg/g)**	32.4	22.8	20.9	18.8	19.1	15.1	18.6	14.8	18.9	13.7
**LE (%)**	97 ± 1.6	110 ± 0.7	110± 0.9	104 ± 1.1	96 ± 1.6	105.4 ± 2.0	98 ± 4.3	109 ± 0.5	103 ± 1.5	105 ± 2.3
**Mean Particle Size (µm)**	5.33	2.48	3.43	7.09	2.94	3.18	2.60	4.24	4.02	3.01
**Span**	2.4	2.9	3.2	3.5	1.1	1.2	1.2	3.3	3.9	1.4

## Supplementary Materials

The following are available online at https://www.mdpi.com/1999-4923/11/10/492/s1, HPLC-ESI-MS/MS method development and validation; Figure S1: Total ion chromatogram of electrospray ionization mass spectrum from purified material and daidzein ion products. Figure S2: Representative MRM chromatograms of blank plasma for daidzein (DZ) (**A**) and for IS (**B**); blank plasma spiked with the analyte at LLOQ (**C**); IS (350 ng/mL) (**D**); plasma samples after oral administration of pure DZ (10 mg/kg) (**E**); and F10 (10 mg/kg) (**F**); Table SI: The effect of hydrophilic polymers, surfactants, and alkalizers with different proportions (0.5, 1, and 2% *w*/*v*) on the solubility of daidzein (DZ) at 37 °C (mean ± S.D, *n* = 3). Table SII: Fitting (*r*^2^) and release constant (K) parameters of mathematical models for daidzein (DZ) release from the solid dispersion (SD) formulations (F1–F10). Table SIII: Intra-day and inter-day precisions and accuracies, extraction recoveries, and the matrix effect for the determination of daidzein (DZ) from the assay samples (mean, *n* = 5).

## References

[1] Fonseca D., Ward W.E. (2004). Daidzein together with high calcium preserve bone mass and biomechanical strength at multiple sites in ovariectomized mice. Bone.

[2] Liu X., Suzuki N., Santosh Laxmi Y.R., Okamoto Y., Shibutani S. (2012). Anti-breast cancer potential of daidzein in rodents. Life Sci..

[3] Aras A.B., Guven M., Akman T., Ozkan A., Sen H.M., Duz U., Kalkan Y., Silan C., Cosar M. (2015). Neuroprotective effects of daidzein on focal cerebral ischemia injury in rats. Neural Regen. Res..

[4] Ajmani P., Yadav H.N., Singh M., Sharma P.L. (2011). Possible involvement of caveolin in attenuation of cardioprotective effect of ischemic preconditioning in diabetic rat heart. BMC Cardiovasc. Disord..

[5] Park M.H., Ju J.W., Park M.J., Han J.S. (2013). Daidzein inhibits carbohydrate digestive enzymes in vitro and alleviates postprandial hyperglycemia in diabetic mice. Eur. J. Pharmacol..

[6] Rothwell J.A., Day A.J., Morgan M.R. (2005). Experimental determination of octanol-water partition coefficients of quercetin and related flavonoids. J. Agric. Food Chem..

[7] Nan G., Shi J., Huang J., Lv J., Yang G., Li Y. (2014). Dissociation constants and solubilities of daidzein and genistein in different solvents. J. Chem. Eng. Data.

[8] Waldmann S., Almukainzi M., Bou-Chacra N.A., Amidon G.L., Lee B.J., Feng J., Kanfer I., Zuo J.Z., Wei H., Bolger M.B. (2012). Provisional biopharmaceutical classification of some common herbs used in Western medicine. Mol. Pharm..

[9] Qiu F., Chen X.Y., Song B., Zhong D.F., Liu C.X. (2005). Influence of dosage forms on pharmacokinetics of daidzein and its main metabolite daidzein-7-*O*-glucuronide in rats. Acta Pharmacol. Sin..

[10] Paudel A., Worku Z.A., Meeus J., Guns S., Van den Mooter G. (2013). Manufacturing of solid dispersions of poorly water soluble drugs by spray drying: Formulation and process considerations. Int. J. Pharm..

[11] Vasconcelos T., Sarmento B., Costa P. (2007). Solid dispersions as strategy to improve oral bioavailability of poor water soluble drugs. Drug Discov. Today.

[12] Yang M., He S., Fan Y., Wang Y., Ge Z., Shan L., Gong W., Huang X., Tong Y., Gao C. (2014). Microenvironmental pH-modified solid dispersions to enhance the dissolution and bioavailability of poorly water-soluble weakly basic GT0918, a developing anti-prostate cancer drug: Preparation, characterization and evaluation in vivo. Int. J. Pharm..

[13] Marasini N., Tran T.H., Poudel B.K., Cho H.J., Choi Y.K., Chi S.C., Choi H.G., Yong C.S., Kim J.O. (2013). Fabrication and evaluation of pH-modulated solid dispersion for telmisartan by spray-drying technique. Int. J. Pharm..

[14] Hengsawas S.S., Keen J.M., Huang S., Zhang F., Mcginity J.W., Williams R.O.I. (2017). Hot melt extrusion versus spray drying: Hot melt extrusion degrades albendazole. Drug Dev. Ind. Pharm..

[15] Ge Y., Wang X., Guo W., Xie X. (2010). Preparation of water-soluble chitosan solid dispersion of daidzein. Zhongguo Zhong Yao Za Zhi.

[16] Feng B.L., Li H.W., Zhou M.Y., Lu W. (2011). Dispersion of daidzein with polyvinylpyrrolidone effects on dissolution rate and bioavailability. Zhong Yao Cai.

[17] Zhang E.J., Ng K.M., Luo K.Q. (2007). Extraction and purification of isoflavones from soybeans and characterization of their estrogenic activities. J. Agric. Food Chem..

[18] Panizzon G.P., Bueno F.G., Ueda-Nakamura T., Nakamura C.V., Dias Filho B.P. (2014). Preparation of Spray-Dried Soy Isoflavone-Loaded Gelatin Microspheres for Enhancement of Dissolution: Formulation, Characterization and in Vitro Evaluation. Pharmaceutics.

[19] Prasain J.K., Jones K., Brissie N., Moore R., Wyss J.M., Barnes S. (2004). Identification of puerarin and its metabolites in rats by liquid chromatography-tandem mass spectrometry. J. Agric. Food Chem..

[20] Xue Y., Hieda Y., Kimura K., Takayama K., Fujihara J., Tsujino Y. (2004). Kinetic characteristics and toxic effects of benzalkonium chloride following intravascular and oral administration in rats. J. Chromatogr. B Analyt. Technol. Biomed. Life Sci..

[21] Ma Y., Zhao X., Li J., Shen Q. (2012). The comparison of different daidzein-PLGA nanoparticles in increasing its oral bioavailability. Int. J. Nanomed..

[22] Cho S.Y., Lee Y.N., Park H.J. (2009). Optimization of ethanol extraction and further purification of isoflavones from soybean sprout cotyledon. Food Chem..

[23] Tran P.H., Tran H.T., Lee B.J. (2008). Modulation of microenvironmental pH and crystallinity of ionizable telmisartan using alkalizers in solid dispersions for controlled release. J. Control. Release.

[24] Paradkar A., Ambike A.A., Jadhav B.K., Mahadik K.R. (2004). Characterization of curcumin-PVP solid dispersion obtained by spray drying. Int. J. Pharm..

[25] Motlekar N., Youan B. (2008). Optimization of experimental parameters for the production of LMWH-loaded polymeric microspheres. Drug Des. Dev. Ther..

[26] Payab S., Davaran S., Tanhaei A., Fayyazi B., Jahangiri A., Farzaneh A., Adibkia K. (2014). Triamcinolone acetonide-Eudragit RS100 nanofibers and nanobeads: Morphological and physicochemical characterization. Artif. Cells Nanomed. Biotechnol..

[27] Sizilio R.H., Galvao J.G., Trindade G.G.G., Pina L.T.S., Andrade L.N., Gonsalves J., Lira A.A.M., Chaud M.V., Alves T.F.R., Arguelho M. (2018). Chitosan/pvp-based mucoadhesive membranes as a promising delivery system of betamethasone-17-valerate for aphthous stomatitis. Carbohydr. Polym..

[28] Kadota K., Otsu S., Fujimori M., Sato H., Tozuka Y. (2016). Soluble hydrolysis-resistant composite formulation of curcumin containing α-glucosyl hesperidin and polyvinylpyrrolidone. Adv. Powder Technol..

[29] Moes J., Koolen S., Huitema A., Schellens J., Beijnen J., Nuijen B. (2013). Development of an oral solid dispersion formulation for use in low-dose metronomic chemotherapy of paclitaxel. Eur. J. Pharm. Biopharm..

[30] Hancock B.C., Parks M. (2000). What is the true solubility advantage for amorphous pharmaceuticals?. Pharm. Res..

[31] Park J.B., Park Y.J., Kang C.Y., Lee B.J. (2015). Modulation of microenvironmental pH and utilization of alkalizers in crystalline solid dispersion for enhanced solubility and stability of clarithromicin. Arch. Pharm. Res..

[32] Hallensleben M.L., Fuss R., Mummy F. (2015). Polyvinyl compounds, others. Ullmann’s Encycl. Ind. Chem..

[33] Najib N.M., Suleiman M., Malakh A. (1986). Characteristics of the in vitro release of ibuprofen from polyvinylpyrrolidone solid dispersions. Int. J. Pharm..

[34] Hu J., Kyad A., Ku V., Zhou P., Cauchon N. (2005). A comparison of dissolution testing on lipid soft gelatin capsules using USP apparatus 2 and apparatus 4. BMC Cardiovasc. Disord..

[35] Meeus J., Lenaerts M., Scurr D.J., Amssoms K., Davies M.C., Roberts C.J., Van Den Mooter G. (2015). The influence of spray-drying parameters on phase behavior, drug distribution, and in vitro release of injectable microspheres for sustained release. J. Pharm. Sci..

[36] Jung H.J., Ahn H.I., Park J.Y., Ho M.J., Lee D.R., Cho H.R., Park J.S., Choi Y.S., Kang M.J. (2015). Improved oral absorption of tacrolimus by a solid dispersion with hypromellose and sodium lauryl sulfate. Int. J. Biol. Macromol..

[37] Seedher N., Kanojia M. (2008). Micellar solubilization of some poorly soluble antidiabetic drugs: A technical note. AAPS PharmSciTech.

[38] Park K. (2015). Drug release mechanisms from amorphous solid dispersions. J. Control. Release.

[39] Varma M.V., Kaushal A.M., Garg S. (2005). Influence of micro-environmental pH on the gel layer behavior and release of a basic drug from various hydrophilic matrices. J. Control. Release.

[40] Brouwers J., Brewster M.E., Augustijns P. (2009). Supersaturating drug delivery systems: The answer to solubility-limited oral bioavailability?. J. Pharm. Sci..

[41] Varma M.V., Kaushal A.M., Garg A., Garg S. (2004). Factors affecting mechanism and kinetics of drug release from matrix-based oral controlled drug delivery systems. Am. J. Drug Deliv..

[42] Asane G.S., Nirmal S.A., Rasal K.B., Naik A.A., Mahadik M.S., Rao Y.M. (2008). Polymers for mucoadhesive drug delivery system: A current status. Drug Dev. Ind. Pharm..

[43] Watanabe S., Yamaguchi M., Sobue T., Takahashi T., Miura T., Arai Y., Mazur W., Wahala K., Adlercreutz H. (1998). Pharmacokinetics of soybean isoflavones in plasma, urine and feces of men after ingestion of 60 g baked soybean powder (kinako). J. Nutr..

[44] Anupongsanugool E., Teekachunhatean S., Rojanasthien N., Pongsatha S., Sangdee C. (2005). Pharmacokinetics of isoflavones, daidzein and genistein, after ingestion of soy beverage compared with soy extract capsules in postmenopausal Thai women. BMC Clin. Pharmacol..

