# Supplementary material for: Manufacturing Different Types of Solid Dispersions of BCS Class IV Polyphenol (Daidzein) by Spray Drying: Formulation and Bioavailability
Source: Pharmaceutics. 2019 Sep 25;11(10):492. doi: 10.3390/pharmaceutics11100492 (PMC6835336; doi:10.3390/pharmaceutics11100492)
Supplement: Supplementary file 1 [file pharmaceutics-11-00492-s001.pdf]

pharmaceutics

Supplementary Materials: Manufacturing Different Types of Solid Dispersions of BCS Class IV Polyphenol (Daidzein) by Spray Drying: Formulation and Bioavailability

Gean Pier Panizzon, Fernanda Giacomini Bueno, Tânia Ueda-Nakamura, Celso Vataru Nakamura and Benedito Prado Dias Filho

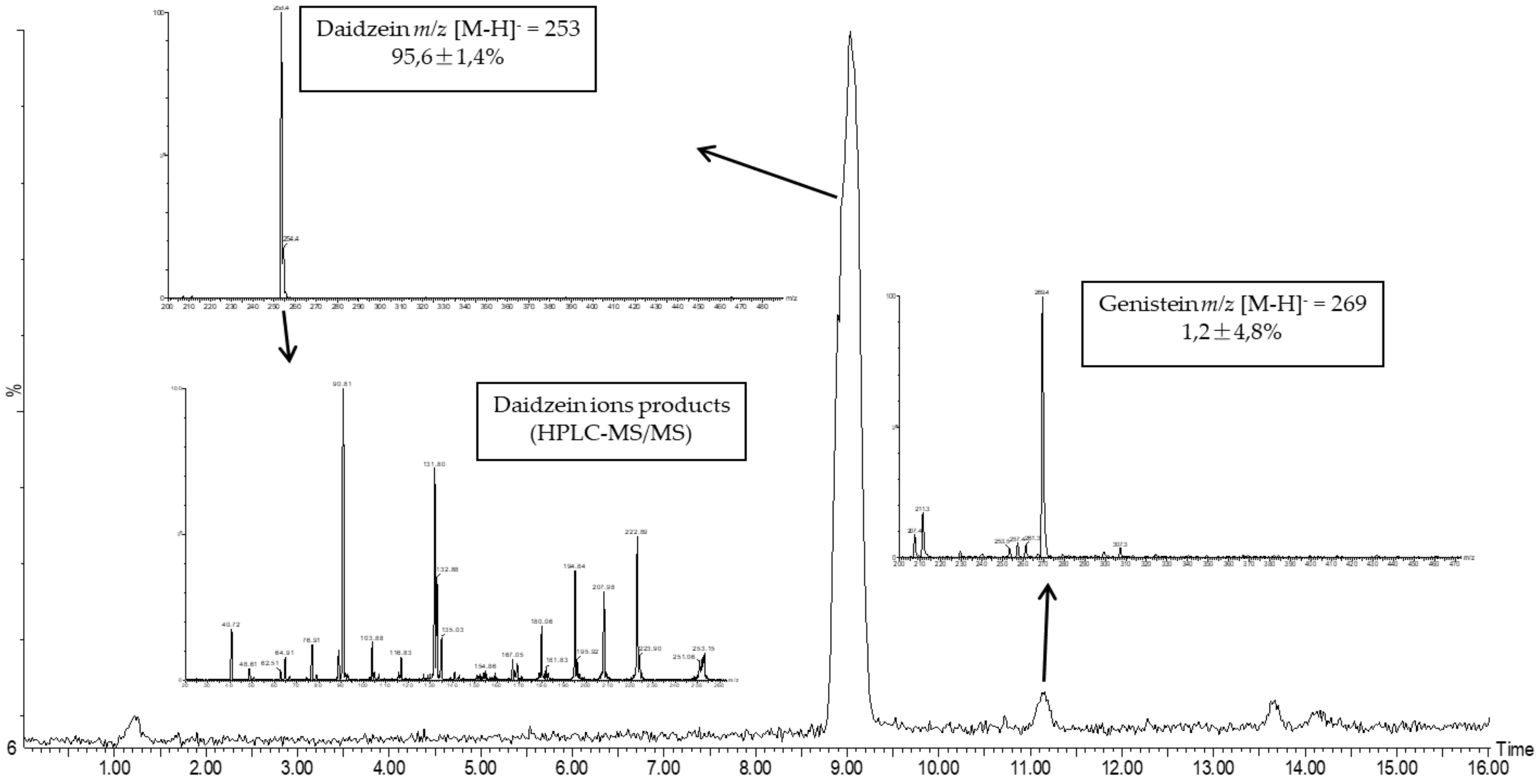

Figure S1. Total ion chromatogram of electrospray ionization mass spectrum from purified material and daidzein ion products.

Table SI. The effect of hydrophilic polymers, surfactants, and alkalizers with different proportions (0.5, 1, and 2% *w/v*) on the solubility of daidzein (DZ) at 37 °C (mean ± S.D, *n* = 3). BAK: benzalkonium chloride; DHP: dipotassium hydrogen phosphate; HPC: hydroxypropyl cellulose; HPMC: hydroxypropylmethylcellulose; PEG: poly(ethylene glycol); PVA: poly(vinyl alcohol); PVP: polyvinylpyrrolidone; SB: sodium borate; Na_2CO_3: sodium carbonate; SCC: sodium carboxymethylcellulose; SDS: sodium dodecyl sulfate; SHC: sodium hydrogen carbonate.

	**DZ (μg/mL)**		
**Hydrophilic polymers**	**0.5% (*w/v*)**	**1% (*w/v*)**	**2% (*w/v*)**
PVP (10 kDa)	3.27 ± 0.1	4.53 ± 0.3	7.27 ± 0.1
PVP (360 kDa)	3.29 ± 0.1	5.34 ± 0.1	8.81 ± 0.1
HPC (80 kDa)	3.32 ± 0.1	3,71 ± 0.0	3.76 ± 0.2
HPC (370 kDa)	2.94 ± 0.1	3.45 ± 0.4	3.87 ± 0.1
HPMC	2.74 ± 0.1	3.33 ± 0,2	4.09 ± 0.1
SCC	1.80 ± 0.2	2.95 ± 0.2	ND
PEG (0.4 kDa)	1.96 ± 0.1	2.08 ± 0.0	2.25 ± 0.1
PEG (2 kDa)	1.96 ± 0.0	2.41 ± 0.0	3.19 ± 0.1
PEG (6 kDa)	1.98 ± 0.0	2.23 ± 0.0	3.01 ± 0.1
Gelatin A	3.10 ± 0.2	3.54 ± 0.4	3.47 ± 0.4
**Surfactants**	**0.5% (*w/v*)**	**1% (*w/v*)**	**2% (*w/v*)**
Pluronic F-68	2.40 ± 0.3	2.80 ± 0.0	4.26 ± 0.3
PVA	2.96 ± 0.0	2.90 ± 0.1	2.81 ± 0.3
Tween 20	9.32 ± 0.1	16.73 ± 0.5	34.67 ± 0.1
Tween 80	7.75 ± 0.2	15.59 ± 0.2	27.20 ± 0.4
SDS	20.20 ± 0.3	46.55 ± 0.3	98.41 ± 3.2
BAK	42.31 ± 2.0	147.85 ± 1.1	317.06 ± 4.4
**Alkalizers**	**0.5% (*w/v*)**	**1% (*w/v*)**	**2% (*w/v*)**
SHC	65.02 ± 2.5	63.63 ± 2.7	51.71 ± 0.8
SB	184.39 ± 2.0	196.23 ± 2.0	211.55 ± 0.5
DHP	69.74 ± 1.8	90.38 ± 1.5	116.42 ± 0.0
Na_2CO_3	3116.79 ± 94.4	4309.59 ± 156.1	5120.47 ± 96.1

ND: Not determined due to high viscosity.

Table SII. Fitting (r^2) and release constant (K) parameters of mathematical models for daidzein (DZ) release from the solid dispersion (SD) formulations (F1–F10).

	**Zero order**		**First order**		**Higuchi**		**Hixson–Crowell**		**Korsmeyer-Peppas**	
	r^2	**K_0 (mg/min)**	r^2	**K_1 (min^{-1})**	r^2	**K_H (mg/min^{½})**	r^2	**K_{HC} (mg$^{1/3}$/min)**	r^2	*n*
**F1**	0.910	0.111	0.940	−0.001	0.986	1.688	0.936	0.002	0.979	0.46
**F2**	0.762	0.260	0.829	−0.004	0.905	4.170	0.808	0.005	0.919	0.56
**F3**	0.773	0.199	0.823	−0.003	0.908	3.174	0.806	0.004	0.923	0.40
**F4**	0.858	0.358	0.944	−0.007	0.964	5.589	0.919	0.008	0.957	0.56
**F5**	0.780	0.461	0.977	−0.022	0.918	7.357	0.948	0.018	0.987	0.63
**F6**	0.992	2.850	0.969	−0.078	0.976	21.99	0.972	0.095	0.993	0.85
**F7**	0.836	0.738	0.989	−0.029	0.939	9.37	0.959	0.027	0.991	0.61
**F8**	0.941	0.581	0.992	−0.018	0.996	8.804	0.939	0.033	0.997	0.61
**F9**	0.868	1.221	0.995	−0.061	0.956	13.08	0.978	0.084	0.989	0.61
**F10**	0.895	0.565	0.997	−0.020	0.983	8.711	0.954	0.032	0.998	0.57

HPLC-ESI-MS/MS Method Development and Validation

To study the oral bioavailability of DZ-loaded SDs, an analytical method of HPLC-ESI-MS/MS was developed and validated.

In the development phase, different additives, including acetic acid (0.1%, *v*/*v*) and formic acid (0.1%, *v*/*v*) with or without ammonium acetate (5–10 mmol), were added into the mobile phase. This was done to optimize LC conditions in order to improve the peak shape, ion count, and shorten the run time. The best results were obtained with formic acid (0.1%, *v*/*v*) as an additive.

In the second step, the ionization modes of DZ and the internal standard (IS) were evaluated. Stronger relative intensity of DZ was seen in positive ion mode than in negative ion mode. Next, pure compounds (500 ng/mL) were individually injected into +ESI by continuous infusion (10–30 µL/min, mixed with 0.5 mL/min mobile phase flow form HPLC) to optimize multiple reaction monitoring (MRM) and MS parameters, including capillary voltage, cone voltage, extraction cone and cone voltage, and collision energy. We used MRM mode in order to develop a specific, analytical method for quantification of DZ in rat plasma.

Various analytical columns were screened, including Kinetex C18 2.6 µm 100A (100 × 2.1 mm, Phenomenex, Torrance, CA, USA), Symmetry C18 3.5 µm (75 × 4.6 mm, Waters, MA, USA), and XTerra MS C18 5 µm (100 × 3.0 mm, Waters, MA, USA) Luna C18(2)-HST 2.5 µm column (50 × 2 mm, Phenomenex, Torrance, CA, USA). The latter provided the best results and was chosen to carry on the quantification. Finally, another approach to develop a reproducible method is the choice of an appropriate internal standard. Initially, genistein and chrysin were tested. Although extraction recoveries of both compounds were appropriated, the relative standard deviation (%RSD) of inter-day precision was too high (>20%). This could be solved by using Glycitein as an IS, which has similar extraction recovery and more importantly, co-elutes with the DZ. These characteristics are desirable because they compensate for the alteration in signal and the matrix effects of the relative efficiency of ionization.

Stock solutions of DZ (10 mg/mL) and IS (8 mg/mL) were prepared by dissolving the accurately weighed reference substances in dimethylsulfoxide. The IS working solution (1000 ng/mL) was prepared in methanol/water (80:20, *v*/*v*). A series of DZ working standard solutions were prepared by diluting the stock solution with methanol/water (80:20, *v*/*v*). The samples for calibrations curves were prepared by spiking blank rat plasma (100 µL) with working solutions of analytes (10 µL) to yield plasma concentrations in the range of 24 to 1505 ng/mL. Quality control (QC) samples at low, medium, and high concentrations (50, 500, and 1000 ng/mL) were also prepared in the same way.

The specificity of the method was investigated by comparing the MRM chromatograms from 5 different rats with those of blank plasma spiked with standard solution and plasma samples after drug administration. This was done in order to identify potential interfering endogenous compounds that may be eluted at the same retention time in DZ and IS. Figure S2 shows the representative MRM chromatograms of blank plasma for DZ (Figure S2A) and IS (Figutr S2B), spiked plasma with the drug at lower limit of quantification (LLOQ) (Figure S2C), internal standard (Figure S2D), and a plasma sample obtained from a rat following oral administration of pure DZ (10 mg/kg) (Figure S2E) and F10 (Figure S2F). The retention times of DZ and IS were found to be 3.60 min. Under the assay condition, no interference from the endogenous plasma or β-glucoronidase was observed, which shows specificity of the method.

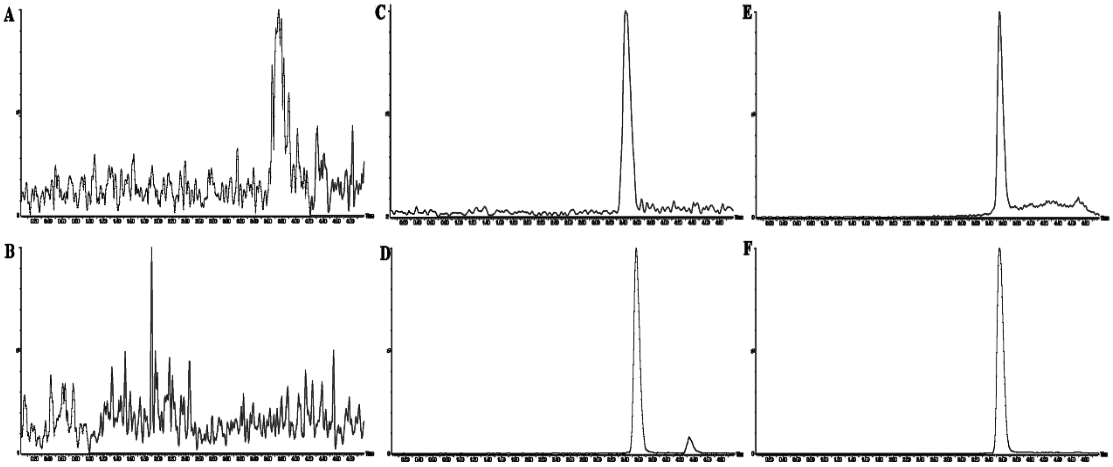

Figure S2. Representative MRM chromatograms of blank plasma for daidzein (DZ) (**A**) and for IS (**B**); blank plasma spiked with the analyte at LLOQ (**C**); IS (350 ng/mL) (**D**); plasma samples after oral administration of pure DZ (10 mg/kg) (**E**); and F10 (10 mg/kg) (**F**).

The linearity of the calibration curve equation was described in the form of $y = ax + b$ (weighing factor $1/x$), where y represents the DZ:IS peak area ratio and x represents the nominal DZ concentration in plasma. The acceptable determination coefficient for a triplicate calibration curve was $r^2 > 0.99$. LLOQ was defined as the lowest concentration of the standard curve, giving a signal-to-noise ratio of 10:1; this can be measured with an acceptable level of precision (RSD < 20%) and accuracy by relative error (RE ± 20%). Linearity of the calibration curve was investigated at six concentration levels (24–1515 ng/mL) by plotting peak area ratio of DZ:IS area (Y-axis)versus the concentration of DZ (X-axis). Good linearity was obtained throughout the concentration range, with the determination coefficient (r^2) within the range of 0.992 to 0.997. The LLOQ was tested with acceptable accuracy and precision until a lower limit of 5 ng/mL was reached.

The intra-day precision was calculated within one day. The inter-day precision was calculated on 2 separate days by analyzing five replicates of QC samples at three different concentrations (24, 505, and 1010 ng/mL). The accuracy was expressed as the relative error (RE %) and the precision as the relative standard deviation (RSD%).

The extraction recoveries of analytes at low, medium, and high concentrations (50, 500, and 1000 ng/mL) were calculated by comparing the peak areas of the extracted (pre-spiked) QC samples with those of the post-spiked standard plasma samples at an equivalent concentration. The recovery of the IS was determined at a single concentration of 350 ng/mL.

$$\text{Recovery (\%)} = (\text{Peak area of pre-spiked sample/Peak area of post-spiked sample}) \times 100 \quad \text{(S1)}$$

The matrix effect of rat plasma constituents over the ionization of DZ at three different concentrations (25, 500, and 1010 ng/mL) and IS (350 ng/mL) were determined by comparing the peak areas of the post-spiked standard plasma samples with those of the neat standard samples at an equivalent concentration.

The inter-day and intra-day precision and accuracy, extraction recovery, and the matrix effect of the analytical method are listed in Table SIII. The intra-day precision and inter-day precision of QC plasma samples (24, 505, and 1010 ng/mL), expressed as relative standard deviation (RSD%), were in the range of 3.48–8.21% and 6.32–14.98%, respectively. The accuracy, calculated as the relative error

(RE%) of inter-day and intra-day at low, medium, and high concentrations, was within the range of −0.66 to 9.02%. All inter- and intra-day precision and accuracy values were acceptable for a bioanalytical method.

With this method, the recovery of the QC samples in this tandem MS method in plasma ranged from 98.74% to 100.00% for DZ, and was 99.49% for IS. Because of the weakly acidic compounds, two acid washes (0.1% acetic acid and 25% methanol + 0.1% acetic acid) were chosen to flush the cartridge. This was done in order to increase the retention in the cartridge, increase the recovery, and eliminate interferents in the sample. Moreover, the optimization of wash solution indicated that 25% methanol could provide a clean background and satisfactory recovery of the samples. The matrix effects derived from QC samples were between 103.56% and 111.45%, and were 99.59% for IS.

Table SIII. Intra-day and inter-day precisions and accuracies, extraction recoveries, and the matrix effect for the determination of daidzein (DZ) from the assay samples (mean, n = 5). IS: internal standard; RSD: relative standard deviation; RE: relative error.

Validation Parameters		DZ (ng/mL)			I.S (ng/mL)
		24	500	1010	350
Intra-day	Precision (RSD, %)	8.21	4.71	3.48	-
	Acuracy (RE, %)	5.6	1.45	−0.59	-
Inter-day	Precision (RSD, %)	14.98	8.72	6.32	-
	Acuracy (RE, %)	9.02	2.91	−0.66	-
Extraction recovery	Mean (%)	100.0	98.74	99.60	99.49
	RSD (%)	1.59	3.65	4.19	3.71
Matrix effect	Mean (%)	111.5	107.0	103.5	99.9
	RSD (%)	1.59	2.45	5.60	3.59

These data indicated that the extraction recovery and matrix effect of the method were acceptable. The validated method was applied to comparative pharmacokinetic studies between pHM-SD (F10) and pure DZ in rat plasma samples obtained after administration of a dose of 10 mg/kg. Three new calibration curves ($r^2 > 0.99$) were created in every batch of samples, and the results were used to calculate concentrations in unknown samples.
